# The Potential of Tissue-Resident Memory T Cells for Adoptive Immunotherapy against Cancer

**DOI:** 10.3390/cells10092234

**Published:** 2021-08-28

**Authors:** Ammarina Beumer-Chuwonpad, Renske L. R. E. Taggenbrock, T. An Ngo, Klaas P. J. M. van Gisbergen

**Affiliations:** 1 Sanquin Research and Landsteiner Laboratory, Department of Hematopoiesis, Amsterdam University Medical Center (UMC), University of Amsterdam, 1066 CX Amsterdam, The Netherlands; R.Taggenbrock@sanquin.nl (R.L.R.E.T.); an_16@live.nl (T.A.N.); k.vangisbergen@sanquin.nl (K.P.J.M.v.G.); 2 Department of Experimental Immunology, Amsterdam University Medical Center (UMC), University of Amsterdam, 1105 AZ Amsterdam, The Netherlands

**Keywords:** adoptive cell therapy, CD8^+^ memory T cells, Cytotoxic T cells, immunotherapy, tissue-resident memory T cells, T cell exhaustion

## Abstract

Tissue-resident memory T cells (T_RM_) comprise an important memory T cell subset that mediates local protection upon pathogen re-encounter. T_RM_ populations preferentially localize at entry sites of pathogens, including epithelia of the skin, lungs and intestine, but have also been observed in secondary lymphoid tissue, brain, liver and kidney. More recently, memory T cells characterized as T_RM_ have also been identified in tumors, including but not limited to melanoma, lung carcinoma, cervical carcinoma, gastric carcinoma and ovarian carcinoma. The presence of these memory T cells has been strongly associated with favorable clinical outcomes, which has generated an interest in targeting T_RM_ cells to improve immunotherapy of cancer patients. Nevertheless, intratumoral T_RM_ have also been found to express checkpoint inhibitory receptors, such as PD-1 and LAG-3. Triggering of such inhibitory receptors could induce dysfunction, often referred to as exhaustion, which may limit the effectiveness of T_RM_ in countering tumor growth. A better understanding of the differentiation and function of T_RM_ in tumor settings is crucial to deploy these memory T cells in future treatment options of cancer patients. The purpose of this review is to provide the current status of an important cancer immunotherapy known as TIL therapy, insight into the role of T_RM_ in the context of antitumor immunity, and the challenges and opportunities to exploit these cells for TIL therapy to ultimately improve cancer treatment.

## 1. Introduction

The potency of the immune system to combat malignancies has been of great interest for the development of novel therapies for cancer patients [1]. Of particular interest are cytotoxic CD8^+^ T lymphocytes that mediate antitumor immunity through recognition of peptide-bound major histocompatibility complex (MHC) class I molecules on the surface of malignant cells. Peptide epitopes for CD8^+^ T cells can arise from neoantigens formed by genome instability of tumor cells [2,3,4,5]. Upon antigen recognition, tumor-specific CD8^+^ T cells have an unsurpassed capacity to eliminate tumor cells through the release of proinflammatory cytokines such as interferon (IFN)-γ and tumor necrosis factor (TNF)-α, and cytotoxic molecules including granzyme B and perforin [6]. However, malignant cells are able to utilize various mechanisms to evade elimination by CD8^+^ T cells. These immune evasion mechanisms include the loss of MHC class I molecule expression on the surface of tumor cells by downmodulating antigen processing and the presentation of peptide antigens on MHC molecules, thereby directly preventing recognition by CD8^+^ T cells [7,8,9]. Another strategy of malignant cells to cripple the immune system is to induce an anti-inflammatory tumor micro-environment (TME). The TME includes a large repertoire of immune cells with immunosuppressive activity, such as tumor-associated macrophages, myeloid-derived suppressor cells and regulatory T (T_REG_) cells. These immune cells are able to dampen effector responses of CD8^+^ T cells through the secretion of anti-inflammatory cytokines, such as IL-4, IL-10 and TGF-β [3,7]. Effector functions and the proliferative capacity of CD8^+^ T cells can also be impaired by the high glycolytic activity of rapidly growing tumor cells resulting in limited availability of glucose for tumor-infiltrating CD8^+^ T cells [10]. The lack of glucose impairs the glycolytic activity in CD8^+^ T cells, which is required for the upregulation of effector functions such as the production of proinflammatory IFN-γ [11]. Moreover, malignant cells can upregulate the metabolic enzyme indoleamine-2,3-dioxygenase (IDO) to limit T cell function via deprivation of the essential amino acids arginine and tryptophan from the TME [12]. Finally, malignant cells and immune cells in the TME upregulate ligands that interact with inhibitory receptors on CD8^+^ T cells to promote immunosuppression and to favor the outgrowth of the tumor [13]. The best characterized inhibitory receptors on tumor-infiltrating lymphocytes (TILs) are programmed cell death protein 1 (PD-1), cytotoxic T lymphocyte associated-antigen 4 (CTLA-4), lymphocyte-activation gene 3 (LAG-3) and T cell immunoglobulin and mucin-domain containing 3 (TIM-3) [14,15,16,17]. Triggering of these receptors induces a state of exhaustion in CD8^+^ T cells resulting in the impaired ability of CD8^+^ T cells to release proinflammatory cytokines [18,19]. The challenge of cancer immunotherapy is to counteract the manipulative strategies that malignant cells utilize to evade elimination through CD8^+^ T cells and other immune cells.

Promising strategies that employ CD8^+^ T cells to fight tumor growth include immune checkpoint blockade therapy and TIL therapy. These therapies reinvigorate antitumor responses of CD8 T cells through direct suppression of inhibitory pathways or through the introduction of greatly expanded numbers of CD8^+^ T cells. However, these therapies currently do not take into account the heterogeneity of the tumor-infiltrating CD8^+^ T cell population. Distinct subsets of CD8^+^ T cells have been identified in in vivo tumor models and in cancer patients. Recently, it has become clear that a large TIL fraction consists of tissue-resident memory T cells (T_RM_). Intratumoral T_RM_ share characteristics with previously identified pathogen-specific T_RM._ These CD8^+^ T cells express adhesion receptors such as CD103 that provide interactions with surrounding tumor cells and downregulate migratory pathways that facilitate entry into the circulation. These characteristics enable T_RM_ to maintain themselves at the tumor site, where they can exert antitumor activities such as the production of proinflammatory cytokines to attract other immune cells or cytotoxic mediators to eliminate tumor cells. Importantly, the presence of intratumoral T_RM_ has been associated with favorable clinical outcomes in various solid cancers [20,21,22,23,24], suggesting that intratumoral T_RM_ may form powerful immunological weapons against tumor growth. Nevertheless, similar to other TILs, intratumoral T_RM_ are exposed to an anti-inflammatory TME and have upregulated expression of inhibitory receptors, which may compromise their ability to clear tumor cells. Therefore, the focal points of our discussion are the challenges and opportunities to apply T_RM_ for immunotherapy. We will focus our discussion on the relevance of T_RM_ for immunotherapy on one important strategy known as TIL therapy that employs TILs to target solid cancers.

## 2. TIL Therapy Is an Important Cancer Immunotherapy

Conventional cancer therapies including surgical resection, radiation therapy, endocrine therapy and chemotherapy have been the standard of care for many decades. These therapies have limitations and are currently insufficient to cure the majority of cancers [7]. A proportion of tumors commonly referred to as ‘hot’ tumors, have a high degree of lymphocyte infiltration and appear to be immunogenic. Therefore, deployment of the host immune system may be a promising strategy to target these hot tumors. Indeed, more recently, several immunotherapies such as chimeric antigen receptor (CAR)-T cell therapy [1,12,25,26], T cell receptor (TCR) gene transfer therapy [1,12,26] and immune checkpoint inhibition [13,27,28,29,30,31] have emerged as successful treatment strategies for cancer patients. In addition to these immunotherapies, TIL therapy has currently also achieved substantial success in the treatment of cancer patients with solid tumors.

TIL therapy utilizes in vitro expanded TILs from resected tumor material for the treatment of cancer patients. TIL therapy was developed based on in vivo experiments showing the antitumor reactivity of in vitro expanded TILs [32,33]. The total lymphocyte fraction at the tumor site was isolated to include tumor-specific T cells in the cultures. After in vitro expansion via anti-CD3-mediated T cell activation in the presence of high doses of IL-2 and reinfusion in tumor-bearing mice, these TILs demonstrated a 50- to 100-fold higher therapeutic potency compared with lymphocyte cultures that were not derived from the tumor [32]. Translation of these mouse studies to human patients have led to promising clinical results in the treatment of metastatic melanoma [34,35,36,37]. Current TIL therapy for melanoma patients employs in vitro expanded TILs originating from resected tumor material for reinfusion into the patient. Using a two-step ex vivo expansion protocol, TILs are initially cultured in the presence of IL-2, before subsequent culture using anti-CD3 and IL-2 in the presence of irradiated allogeneic feeder cells [38,39,40]. Exogenous IL-2 is supplied during these cultures to reinvigorate exhausted T cells that were extracted from the tumor tissue [41,42]. Clinical studies have shown that TIL therapy is highly effective and results in objective response rates of up to 50%, and complete remission in 10–20% of patients with metastatic melanoma [35,43,44,45]. The success in the treatment of end stage melanoma patients has opened doors for adoptive cell therapy employing TILs in the fight against several other types of cancers, such as cervical carcinoma [46], breast carcinoma [47] and non-small-cell lung carcinoma [48,49].

Despite these clinical successes, improvements of TIL therapy are required to further optimize the treatment options of cancer patients. TIL therapy is a personalized therapy that employs expanded T cells from resected tumor material from the patient. The strict dependence on T cells of the patient results from their HLA restrictions. T cells recognize antigens in the context of HLA molecules, which are highly polymorphic, limiting the utility of T cells between different individuals. The importance of limiting patient material for TIL therapy suggests that strategies that reduce cell number requirements will benefit therapeutic options. Currently, TIL therapy requires large numerical expansion to generate the more than 1 × 10^10^ TILs required for reinfusion into the patient to counter tumor growth [38]. TIL expansion protocols are lengthy and laborious to achieve these cell numbers. Moreover, the introduction of a large number of donor T cells in TIL therapy also present challenges for the availability of homeostatic cytokines, which are crucial for the persistence of T cells under steady state conditions. The homeostatic cytokines IL-7 and IL-15 in recipients of adoptive T cell therapy are essential to support the survival of donor T cells after reinfusion. However, donor T cells have to compete with host cells for the limited availability of these homeostatic cytokines. Lymphodepletion prior to TIL infusion maximizes the potential of the adoptively transferred cells through removal of competing host T cells [50]. Lymphodepletion also augments TIL efficacy through transient elimination of suppressive CD4^+^CD25^+^ T_REGS_ and increased activity of antigen-presenting cells to stimulate donor T cells [40,51]. However, lymphodepletion protocols have disadvantages such as collateral damage to the recipient tissues. Therefore, more sophisticated strategies to improve the efficiency of TIL therapy are required.

Effective TIL therapy is dependent on the presence of endogenous tumor-specific T cells in the tumor. However, recent studies have shown that the majority of tumor-infiltrating T cells are bystanders that do not recognize tumor antigens [52,53]. Therefore, improvement of TIL therapy may be achieved through selection of tumor-specific T cells within the donor T cell pool [40,50]. An alternative strategy to improve TIL therapy may be through selection of tumor-specific T cells with optimal capacity to counter tumor growth. The exhausted phenotype of a large proportion of TILs suggests that room for improvement may exist in the selection of functional T cells at the tumor site. The strong association of T_RM_ with increased survival of cancer patients suggests that these T cells are prime candidates for selection into TIL therapy. We will next discuss the differentiation pathways of T cells after tumor development. This information is essential to address a major future challenge of TIL therapy on how to achieve the selective expansion of tumor-specific T cells and of T cells with optimal ability to counter tumor growth.

## 3. Development of T Cell Exhaustion in the Tumor Microenvironment

Efforts to improve T cell-dependent immunotherapies against cancer start with a better understanding of T cell differentiation in a tumor setting. Tumors create an environment in which T cells are persistently activated with antigens, thereby triggering these T cells to enter a distinct differentiation pathway resulting in T cell exhaustion [54]. Exhausted T (T_EX_) cells have been described in melanoma [18,55], ovarian carcinoma [16], hepatocellular carcinoma [56], urothelial carcinoma [57], pancreatic carcinoma [58], and non-small-cell lung carcinoma [59]. T_EX_ cells form a lineage with a unique epigenetic and transcriptional profile distinct from that of memory T cells arising after acute infection [54]. In contrast to these memory T cells that survive independent of cognate antigen and undergo self-renewal driven by the homeostatic cytokines IL-7 and IL-15, T_EX_ cells require persistent antigenic stimulation [60]. Therefore, it is not unexpected that antitumor T cells exhibit similar characteristics to virus-specific T cells in chronic infections [61,62,63]. In fact, T_EX_ cells have first been described in the lymphocytic choriomeningitis virus (LCMV) Clone 13 infection model, which similarly to tumors, induces persistent antigenic stimulation [64,65]. More recently, T_EX_ cells have been observed in human infections, including human immunodeficiency virus (HIV) [66,67,68], hepatitis B and C viruses (HBV/HCV) [69,70].

T cell exhaustion is identified by the progressive loss of effector functions, in particular, the production of proinflammatory cytokines and by the sustained expression of inhibitory receptors that suppress T cell activity [41,54]. T cell exhaustion is a differentiation process under the control of transcription factors including TOX, BLIMP-1, EOMES and NR4A that regulate their effector function and the expression of inhibitory receptors [71]. Persistent antigen stimulation and inflammation are thought to drive the sequential loss of effector functions. Loss of IL-2 production is the earliest sign of exhaustion [72,73]. Next, TNF-α production can become compromised [72,73]. IFN-γ production has shown to be more resistant to exhaustion, but is ultimately lost after chronic inflammation [72,73]. T_EX_ cells may undergo these adaptations to reduce immunopathology, as they potentially cause major tissue damage by secreting proinflammatory cytokines [74,75]. While the production of cytokines is sequentially lost, T_EX_ cells appear to maintain the expression of chemokines including CCL3 (MIP-1α), CCL4 (MIP-1β) and CXCL10 (IP-10) [76]. Exhausted CD8^+^ T cells may also maintain cytotoxic function, given that they have been shown to constitutively produce high levels of granzyme B [63]. The persistence of partial effector function in T_EX_ appears to be functionally relevant in combatting tumor growth.

T_EX_ upregulate inhibitory receptors, which function as immune checkpoints that limit immune activation and prevent autoimmunity [77,78]. Inhibitory receptors that have been associated with T cell exhaustion include PD-1, CTLA-4, LAG-3, TIM-3, CD38, CD39, CD160, 2B4 and TIGIT [79]. PD-1 is the most prominent inhibitory receptor associated with T cell exhaustion [41]. PD-1 is readily upregulated upon T cell activation and its expression persists on T_EX_ [80]. PD-1 recognizes its ligand PD-L1, which is often expressed on tumor cells, and PD-L2, which is present on dendritic cells and macrophages, allowing these cells to employ interactions with inhibitory PD-1 to dampen T cell responses [81]. PD-1 carries an intracellular tail containing an immunotyrosine inhibitory motif (ITIM) and an immunotyrosine switch motif (ITSM), which can recruit phosphatases that dephosphorylate key signal transducers, thereby preventing engagement of proximal signaling molecules with the TCR [82] as well as on the costimulatory molecule CD28 [83,84]. In this manner, PD-1 signaling reduces T cell activation, proliferation, and cytokine secretion of T_EX_ [81]. Therefore, blockade of PD-1 or PD-L1 may lead to reinvigoration of T_EX_ cells and the establishment of robust antitumor responses. Thus, blockade of PD-1 and other inhibitory receptors on tumor-specific T_EX_ cells appears to be an effective therapeutic strategy to reinvigorate T_EX_ cells to counter tumor growth. Taken together, although T_EX_ cells may be interesting therapeutic targets for cancer immunotherapy, the reinvigoration of these T cells into fully functional T cells appears a necessity to boost antitumor responses.

## 4. Exhausted T Cell Subsets in Tumor Tissue

**Figure 1:**
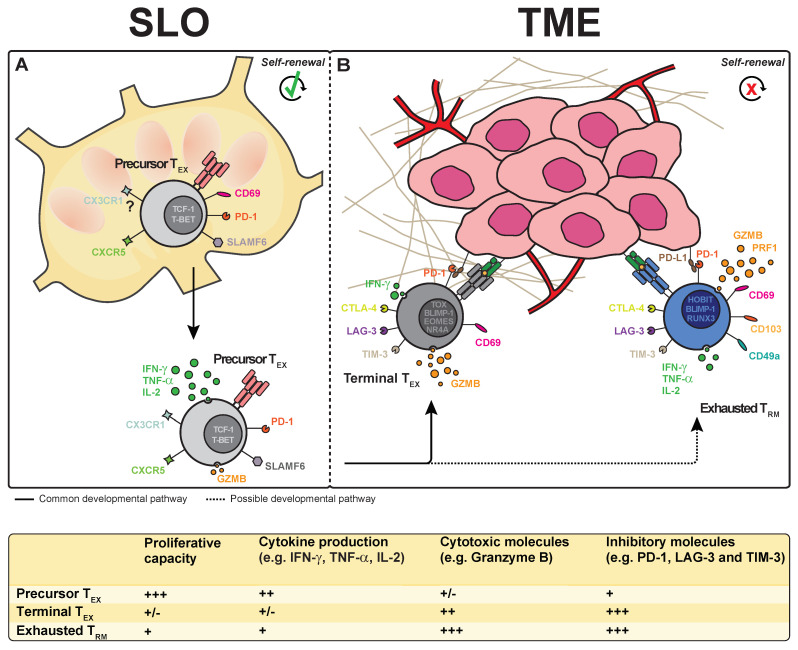
The differentiation pathway of exhausted T cells and tissue-resident memory T cells in the tumor microenvironment. (**A**) Upon activation, precursor exhausted T (T_EX_) cells expressing the surface molecules SLAMF6, CXCR5 and CD69 and the transcription factors TCF-1 and T-BET migrate from the T cell zones of the secondary lymphoid organs (SLO) towards the tumor microenvironment (TME). (**B**) In the TME, precursor T_EX_ differentiate into terminal T_EX_, which express the transcription factors TOX, BLIMP-1, Eomes and NR4A, and have an impaired ability to produce cytokines (e.g., IFN-γ, TNF-α and IL-2), but an increased production of cytotoxic molecules (e.g., granzyme B). Terminal T_EX_ also upregulate the expression of inhibitory receptors, such as PD-1, CTLA-4, LAG-3 and TIM-3. Precursor T_EX_ may also give rise to intratumoral tissue-resident memory T (T_RM_) cells expressing the transcription factors BLIMP-1, HOBIT and RUNX3 and the extracellular molecules CD69, CD103 and CD49a. Similar to terminal T_EX_, T_RM_ upregulate inhibitory receptors and downregulate cytokine responses. In contrast, T_RM_ appear to maintain expression of cytotoxic molecules. Abbreviations: BLIMP-1, B lymphocyte-induced maturation protein 1; CTLA-4, cytotoxic T-lymphocyte-associated protein 4; CX3CR1, CX3C chemokine receptor 1; CXCR5, C-X-C chemokine receptor type 5; GZMB, granzyme B; HOBIT, homolog of Blimp-1 in T cells; IFN-γ, interferon gamma; IL-2, interleukin 2; LAG-3, lymphocyte-activation gene 3; NR4A, nuclear hormone receptor 4A; PD-1, programmed cell death protein 1; PRF1, perforin 1; RUNX3, runt-related transcription factor 3; SLAMF6, SLAM family member 6; T-BET, T-box transcription factor 21; TCF-1, T-cell factor 1; TIM-3, T-cell immunoglobulin and mucin domain 3; TNF-α, tumor necrosis factor alpha; TOX, thymocyte selection-associated high-mobility group box protein.

Compelling evidence shows that the T_EX_ population is heterogeneous and consists of different subsets. The majority of T_EX_ cells appear terminally differentiated, display a SLAMF6^low^TCF-1^low^CXCR5^low^EOMES^high^PD-1^high^ phenotype and have low proliferative potential (Figure 1) [41]. In contrast to these terminal T_EX_, a numerical minority of T_EX_ is characterized by SLAMF6^high^TCF-1^high^CXCR5^high^T-BET^high^PD-1^int^ expression. This subset of T_EX_ displays high proliferative potential and predominantly localizes to lymphoid tissue rather than the tumor site, where terminal T_EX_ mainly reside (Figure 1A). The lymphoid tissue could provide a protective niche for this minor T_EX_ population away from the immunosuppressive environment of the tumor site [85]. In line with evidence from in vivo tumor models, it has been proposed that this fraction forms T_EX_ precursors that can maintain the terminal T_EX_ population (Figure 1B) [63,85]. Importantly, the increased frequency of T_EX_ precursors is associated with an improved clinical outcome for cancer patients. Moreover, immune checkpoint blockade therapies result in an increased amount of T_EX_ precursors that boost the T cell response against the tumor [63,71,86,87]. Thus, T_EX_ precursors appear to be a more attractive subset for immunotherapy of cancer patients than terminal T_EX_.

Recent studies also suggest the presence of T cells displaying a phenotype resembling that of T_RM_ in tumor tissues of cancer patients. Tumor infiltrating T cells with T_RM_-like characteristics have been described in several human cancers, including melanoma [20,88], endometrial adenocarcinoma [23], lung cancer [49,89,90,91,92], bladder cancer [93,94], ovarian cancer [21,95,96], cervical cancer [97], breast cancer [22,98] and colorectal cancer [99]. T_RM_ have initially been identified in acute infection models as a lineage that is distinct from circulating memory subsets, including central memory T (T_CM_) cells and effector memory T (T_EM_) cells. Under steady state conditions, T_RM_ cells are permanently maintained in peripheral tissues without accessing the bloodstream, in contrast to circulating T_CM_ and T_EM_ that patrol secondary lymphoid organs and peripheral tissues, respectively [100,101]. Although T_RM_ persist in the peripheral tissues during homeostasis, they are able to exit these tissues after antigenic or inflammatory stimulation, such as occurs during reinfection [102,103]. Thus, it is not inconceivable that tumor T_RM_ may have access to the bloodstream in the presence of persistent antigens, such as occurs in a tumor setting.

The main phenotypic characteristics to distinguish T_RM_ cells from their circulating counterparts include the expression of extracellular markers, such as the C-type lectin CD69, the αE integrin CD103 and the VLA-1 subunit CD49a [104,105]. These molecules provide essential contributions for the persistence of T_RM_ in the tissues. CD69 captures T_RM_ in the peripheral tissues through suppression of S1PR1-driven tissue exit in response to the chemoattractant S1P in blood and lymph [106,107]. CD103 is an integral component of the αEβ7 integrin that mediates adhesion to E-cadherin on epithelial cells [108,109]. In addition, CD49a is an integrin component that allows T_RM_ to anchor into the extracellular matrix through binding of collagens [110]. T_RM_ also express and utilize a distinct set of transcription factors including RUNX3, HOBIT, BLIMP-1 and NOTCH that regulate their tissue residence and effector functions [104,105,111]. These characteristics distinguish T_RM_ from circulating memory T cells that develop in acute infection. Tumor T_RM_ share many surface molecules including CD69, CD103 and CD49a with pathogen-specific T_RM_, although their expression may vary between T_RM_ in different tumor types [24]. It is less clear how well these molecules identify T_RM_ from other T cell subsets arising in a tumor setting. The definitions of tumor T_RM_ have not yet been clearly demarcated to separate them from populations of T_EX_. For example, tumor T_RM_ may share expression of CD69 with subsets of precursor and terminal T_EX_ [112]. It is of importance to note that tumor-resident T_RM_ can be clearly identified as a separate population from other T cell subsets based on their CD103 expression [89,90,96,99,113,114]. Transcriptional analysis of CD103^+^ T cells in lung carcinoma and in head and neck squamous carcinoma have shown that these cells appear to genuinely represent T_RM_, based on other characteristics such as lack of tissue exit receptors such as S1PR1 [113]. However, the overlay of the current classifications of circulating T cells versus T_RM_ and precursor versus terminal T_EX_ requires further research.

T_RM_ in skin, lungs, female reproductive tract and at other sites have been established as essential immune cells in the protection against reinfection in different experimental infection models [115,116,117,118,119]. Their strategic location at prime entry sites of pathogens in the epithelial and mucosal tissues as well as their potential to immediately respond with the production of proinflammatory cytokines may contribute to the superior potential of T_RM_ in protection against reinvading pathogens [100,120,121]. Moreover, the persistence of T_RM_ in the peripheral tissues, which may depend on the presence of homeostatic cytokines such as IL-7 and IL-15, ensures long-term protection against reinfection [122,123]. The importance of T_RM_ cells in protection against secondary infection with acute viruses [102,124] has sparked the interest for their role in tumor control. Underlining a protective role of T_RM_ against tumor growth, the prevalence of these T cells in tumor tissue has been associated with favorable clinical outcomes in several cancer types, among which are breast cancer, bladder cancer, lung cancer, cervical cancer, colorectal cancer, gastric cancer, ovarian cancer, melanoma and endometrial adenocarcinoma [20,21,22,23,89,97,98,125,126,127,128]. The frequency of T_RM_ appears to outperform the total T cell count in the tumor as a prognostic marker in these cancer patients [89,126,129]. However, the frequency of T_RM_ cells was not able to predict survival of patients suffering from pancreatic cancer [130], suggesting that T_RM_ may not be protective against all cancer types. Nevertheless, these findings highlight the presence and relevance of CD103^+^ T_RM_ in tumors for the majority of cancer types.

Tumor-infiltrating T_RM_ do not appear to control tumor growth through the production of proinflammatory cytokines. IFN-γ, TNF-α and IL-2 expression were relatively decreased in tumor-derived T_RM_ cells compared with circulating T cells in melanoma patients [88]. Similarly, IFN-γ and TNF-α production were decreased in CD103^+^ tumor-infiltrating T cells compared with other T cell subsets in head and neck squamous cell carcinoma [131]. In contrast, T_RM_-like cells found in endometrial and breast cancer retained equal capacities to produce IFN-γ, TNF-α and IL-2, compared with tumor-infiltrating T cells that did not display a T_RM_ phenotype [126,132]. Relative to other tumor-infiltrating T cells, CD103^+^ T_RM_ also displayed upregulation of immune checkpoint receptors such as PD-1, LAG-3, CTLA-4 and TIM-3 (Figure 1B) [20,88,90,133]. Differences may exist between T_RM_ populations, given that T_RM_ extracted from NSCLC and melanoma do not express CTLA-4 [20,89] and T_RM_ originating from ovarian cancer only weakly express CTLA-4, TIM-3 and LAG-3 [127]. The expression of these inhibitory receptors suggests that the majority of tumor T_RM_ display an exhausted phenotype and that these memory T cells may be reinvigorated using immune checkpoint inhibition therapies. In fact, immune checkpoint blockade of PD-1 and TIM-3 appears to enhance T_RM_-driven cytokine production [89,134,135]. Furthermore, anti-PD-1 blockade in both a melanoma mouse model, as well as in patients receiving anti-PD-1 therapy, increased the numbers of intratumoral T_RM_ cells [88,136]. These findings imply that inhibitory receptors on T_RM_ may restrain T_RM_-driven antitumor responses and that relief of their suppression may enhance the therapeutic potential of T_RM_. Their decreased cytokine production and increased expression of inhibitory receptors also indicate that T cells defined as T_RM_ in these tumors overlap with a fraction of the T_EX_ population.

The compromised cytokine responses of tumor-infiltrating T_RM_ suggest that these cells employ different effector pathways to counter tumor growth. Indeed, tumor-resident T cells appear well-equipped to eliminate tumor cells through the release of cytotoxic molecules. Transcripts of cytotoxic effector molecules granzyme A and B were found to be upregulated in CD103^+^ T cells that exerted a T_RM_ phenotype in lung carcinoma patients [90]. Moreover, CD103^+^ T_RM_-like T cells expressed perforin and granzymes A and B at protein level, in contrast to the CD103^−^ fraction of tumor-infiltrating T cells [22,89,90,94]. CD103^+^ T cells were also more efficient in killing autologous tumor cells, compared with their CD103^−^ counterparts, as was demonstrated using in vitro co-cultures [89,131]. These studies suggest that the enhanced expression of cytotoxic molecules endows tumor T_RM_ with superior killing abilities to maintain control of tumor growth. Taken together, tumor-associated T_RM_ appear to maintain high expression of cytotoxic molecules, whereas their ability to produce proinflammatory cytokines to counter tumor growth might be restrained through the expression of inhibitory receptors. These findings indicate that, similar to precursor T_EX_ cells, T_RM_ cells are an attractive target for cancer immunotherapy.

## 5. T Cell Subsets in TIL Therapy

**Figure 2:**
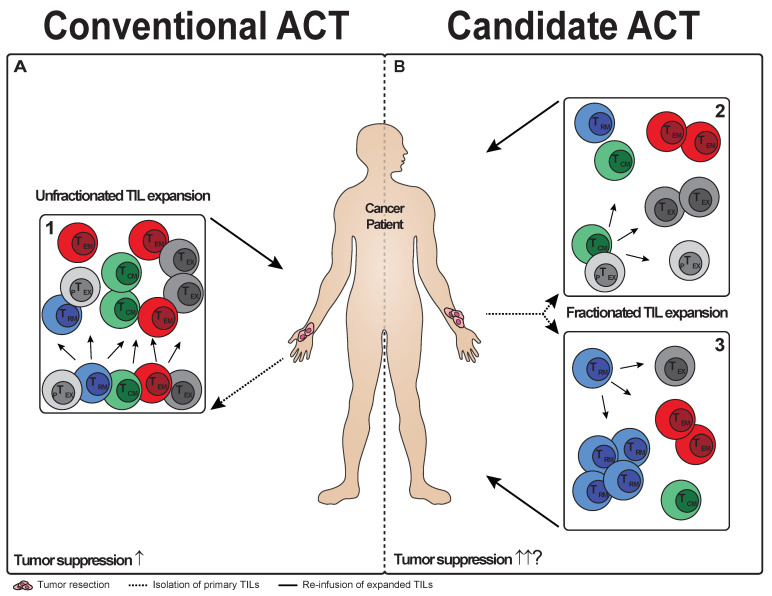
Strategies of conventional and candidate adoptive T cell therapy. (**A**) TIL therapy involves the isolation and expansion of tumor-infiltrating lymphocytes (TILs) from tumor tissue for reinfusion into the cancer patient. The current strategy employs unfractionated TILs that may include central memory T (T_CM_) cells, effector memory T (T_EM_) cells, tissue-resident memory T (T_RM_) cells and precursor and terminal exhausted T (T_EX_) cells (panel 1). (**B**) A potential novel strategy of TIL therapy is to select T_CM_ or precursor T_EX_, which have high potential to form the complete spectrum of T cell subsets. However, these precursor cells may have limited potential to form T_RM_ (panel 2). Therefore, another approach to establish improved TIL therapy may be to select T_RM_ cells from tumor tissue, which have intrinsic capacity to reform T_RM_ (panel 3). Both strategies may have the potential to improve the efficacy of TIL therapy to counter tumor growth.

Adoptive cellular therapies such as TIL exploit the antitumor potential of the immune system and have shown promising clinical results in the regression of various tumors. However, despite the substantial progress that has been made, only a fraction of treated patients achieves durable responses, suggesting room for improvement of these cellular therapies. Current TIL therapies make use of unfractionated T cell populations for in vitro expansion to generate large quantities of T cells with antitumor potential. These unfractionated T cells extracted from the tumor are heterogeneous in respect to their specificity and their differentiation stage (Figure 2A). In fact, the majority of CD8^+^ tumor-infiltrating T cells do not recognize tumor antigens and are considered bystander cells without an apparent direct role in establishing tumor clearance. To a large degree, such bystander T cells in tumors have the phenotype of T_RM_, as indicated by co-expression of CD69 and CD103 [137]. A proportion of these intratumoral, but non-tumor-responsive T cells have been shown to recognize viruses [52,138]. Interestingly, reactivation of these intratumoral virus-specific CD8^+^ T cells via local injection of viral peptides induced an immunostimulatory environment within the tumor, resulting in delay of tumor growth [137]. This supports the notion that tumor-residing T_RM_ can contribute to tumor clearance upon adequate stimulation with cognate antigen.

Despite high phenotypic overlap with tumor-specific T cells, bystander T cells lack surface expression of CD39 and 4-1BB. These receptors have been identified as TCR-induced molecules that are preferentially expressed on tumor-reactive T cells in several solid cancers [52,139]. These surface molecules may enable selection of tumor-reactive TILs to improve the response rate of donor T cells in adoptive T cell therapy [52,53,139,140]. Therefore, improvement of TIL therapy may be achieved through selection of tumor-specific CD8^+^ T cells with optimal capacity to counter tumor growth.

Deletion of undesirable T cell subsets or selection of desirable T cell subsets for in vitro expansion may also maximize the therapeutic potential of adoptive TIL therapy (Figure 2B). Regulatory T cells have been found to accumulate in tumor tissue relative to peripheral blood [141]. These cells have the ability to suppress antitumor responses of T cells and therefore constitute an undesirable T cell subset in the TIL product. Therefore, the selective removal of CD4^+^ T cells that includes the complete fraction of regulatory T cells may improve the effectiveness of the TIL product. Not only deletion of counter-effective T cells from the TIL product, but also selection to allow the specific outgrowth of T cell subsets with an optimal ability to counter tumor growth may improve TIL therapy. The capacity of specific memory CD8^+^ T cell subsets to eliminate tumor cells has been addressed in experimental settings of adoptive cellular therapy. Adoptively transferred populations of tumor-specific T_CM_ and T_EM_ have been shown to give rise to effector responses that suppressed tumor growth in tumor-bearing mice. However, responses originating from T_CM_ demonstrated superior antitumor activity compared with those originating from T_EM_ [142]. The underlying reason for the efficacy of T_CM_ cells in countering tumor growth may relate to their superior in vivo proliferative capacity and their ability to induce recall responses [143,144,145,146,147]. T_CM_ are able to generate secondary T_CM_ ensuring self-renewal and persistence of the adoptively transferred memory cells. They are also able to differentiate into T_EM_ and effector T cells, which have robust abilities to eliminate tumor cells [148]. In contrast, T_EM_ are restricted in their potential to form secondary responses of effector T cells [149,150]. These findings designate T_CM_ as superior candidates for fractionated adoptive cell therapies compared with T_EM_. Characterization of TILs in solid tumors, such as prostate carcinoma, lung carcinoma and melanoma has shown that they are dominated by subsets of exhausted T cells distinct from T_CM_ and T_EM_ [47,151]. Intratumoral T_EX_ are maintained by precursor T_EX_, which similar to T_CM_, have high self-renewal and repopulation potential, suggesting that these T_EX_ are superior in countering tumor growth upon adoptive transfer. Since the majority of precursor T_EX_ reside in lymphoid tissue, only a minor fraction of these cells will be retrieved from the tumor in the TIL product for adoptive transfer into cancer patients. Nevertheless, exploring selective employment of precursor T_EX_ appears to be relevant for tumor immunotherapy.

Tumor-infiltrating T_RM_ are associated with improved tumor growth control, suggesting that the successful implementation of these T cells in cellular therapy may benefit treatment of cancer patients. However, the current nonselective culture protocols position T_RM_ at a disadvantage relative to other tumor-derived T cells for inclusion in the TIL product. The antigen non-specific expansion of unfractionated TILs using anti-CD3 antibodies and IL-2 suggests that fast growing subsets can outcompete slow growing subsets in the culture. These differential growth rates of distinct T cell subsets in the tumor may result in the omission of T_RM_ from the final TIL product. Indeed, it appears that CD103^+^ T_RM_-like TILs underwent fewer rounds of proliferation compared with their CD103^−^ counterparts upon culture in IL-2 [133]. These findings suggest that current regimens for TIL expansion may result in a substantial reduction of CD103^+^ T_RM_-like cells in the final TIL product. Despite their competitive disadvantage in current TIL cultures, T_RM_ possess a considerable proliferative capacity. In response to antigenic challenge, T_RM_ have been shown to substantially contribute to both local and systemic secondary T cell responses [102,124]. Importantly, T_RM_ can achieve durable repopulation of local T_RM_ pools after restimulation [102,124,149,152,153,154]. Previous reports have also demonstrated that T_RM_ from various tissues can be expanded in culture [133,155]. It is possible that current culture protocols are not yet optimized for the expansion of T_RM_. Standardized culture regimens provide glucose-rich media to expand T cells. However, evidence suggests that T_RM_ mainly rely on mitochondrial β-oxidation of exogenous free fatty acids (FFA) to persist long-term in the peripheral tissues [156]. FFA uptake is regulated by the increased expression of fatty acid binding protein (FABP)4 and FABP5 on T_RM_ relative to circulating memory T cells [156]. Although it is currently unclear how these findings apply to intratumoral settings, they suggest an opportunity to improve expansion of T_RM_ in culture for the purpose of immunotherapy.

The inclusion of T_RM_ in the TIL product may even require selective outgrowth of T_RM_, given that strong evidence suggests that other T cells have an impaired potential to induce CD103^+^ T_RM_ [149]. Recent studies have shown that in contrast to naïve T cells, T_CM_ are compromised in their potency to develop into T_RM_ in the skin upon restimulation [136]. Similarly, we and others have reported that T_CM_ were unable to give rise to CD103^+^ T_RM_ cells at mucosal sites including the skin and small intestine [149,157]. In contrast to naïve T cells, T_CM_ and T_EM_ are unable to robustly upregulate CD103 expression upon stimulation with TGF-β. The inability of circulating T cell subsets to upregulate CD103 in response to TGF-β signaling may be attributed to differential epigenetic imprinting of the *Itgae* locus [149]. Chromatin accessibility of the *Itgae* locus encoding CD103 was found to be higher in naïve T cells compared with circulating memory T cells [149,158,159]. In particular, the accessibility of binding regions for RUNX and SMAD transcription factors, which are key targets of TGF-β signaling, was higher in naïve T cells compared with circulating memory T cells [160,161]. Given that circulating memory T cells are unable to induce CD103^+^ T_RM_, strategies selectively employing T_RM_ for expansion seem relevant to develop these memory T cells for immunotherapy.

Challenges remain in the development of T_RM_ for cellular adoptive therapies, such as their relocation into tumor tissue following reinfusion in the bloodstream. T_RM_ take permanent residence in the tissues and do not access the bloodstream. Therefore, it is uncertain whether T_RM_ maintain the machinery that is required to access the tumor site after injection into the bloodstream. Reports showing that T_RM_ cells are predisposed to home to their original tissue sites upon transfer suggest that T_RM_ maintain the ability to relocate from the bloodstream into the tissues [124,162,163]. Additionally, intratumoral delivery of expanded T_RM_ cells may be an alternative approach to reinfuse these cells. The injection of DCs into the tumor site has previously been proven effective [164,165,166], but it is unclear whether this strategy is feasible for T_RM_. Taken together, despite these hurdles, T_RM_ cells appear promising candidates for employment in tumor eradication. T_RM_ cells are able to undergo multiple rounds of proliferation after restimulation and exert robust effector responsiveness [167]. These characteristics of T_RM_ may be highly beneficial for persistence at sites where chronic stimulation might occur, such as in tumor settings. However, further investigation is crucial to elucidate the full potential of T_RM_ for adoptive transfer therapy to eradicate solid tumors.

## 6. Concluding Remarks

The deployment of immune cells in the fight against cancer has become of great interest in the past years. TIL therapy has shown promise in the treatment of different cancer types. However, durable responses are not achieved in a large fraction of cancer patients, indicating that further improvement of this T cell-driven therapy is required. An area of intense investigation is the differentiation pathway of T cells in a tumor setting. Distinct subsets of precursor T_EX_, terminal T_EX_ and T_RM_ have been characterized from resected tumor material and in in vivo tumor models. In particular, precursor T_EX_ and T_RM_ have been strongly associated with improved survival of cancer patients [20,23,89,90,94,97]. Thus, the fractionation of T cells into subsets, in particular the enrichment of T cell preparations for precursor T_EX_ or T_RM_, may boost the potential of current TIL-centered therapies.

## Data Availability

Not applicable.
